# Supplementary material for: Critical evaluation of an autologous peripheral blood mononuclear cell-based humanized cancer model
Source: PLoS One. 2022 Sep 12;17(9):e0273076. doi: 10.1371/journal.pone.0273076 (PMC9467357; doi:10.1371/journal.pone.0273076)
Supplement: S1 Fig — Schematic showing the workflow and the time frame of humanized Pancreatic and Colorectal cancer iPDX mouse model establishment and analysis of T cell engraftment. (PDF) [file pone.0273076.s001.pdf]

S1 Fig. Development of a humanized mouse model (iPDX). Schematic showing the workflow and the time frame of humanized Pancreatic and Colorectal cancer iPDX mouse model establishment and analysis of T cell engraftment.

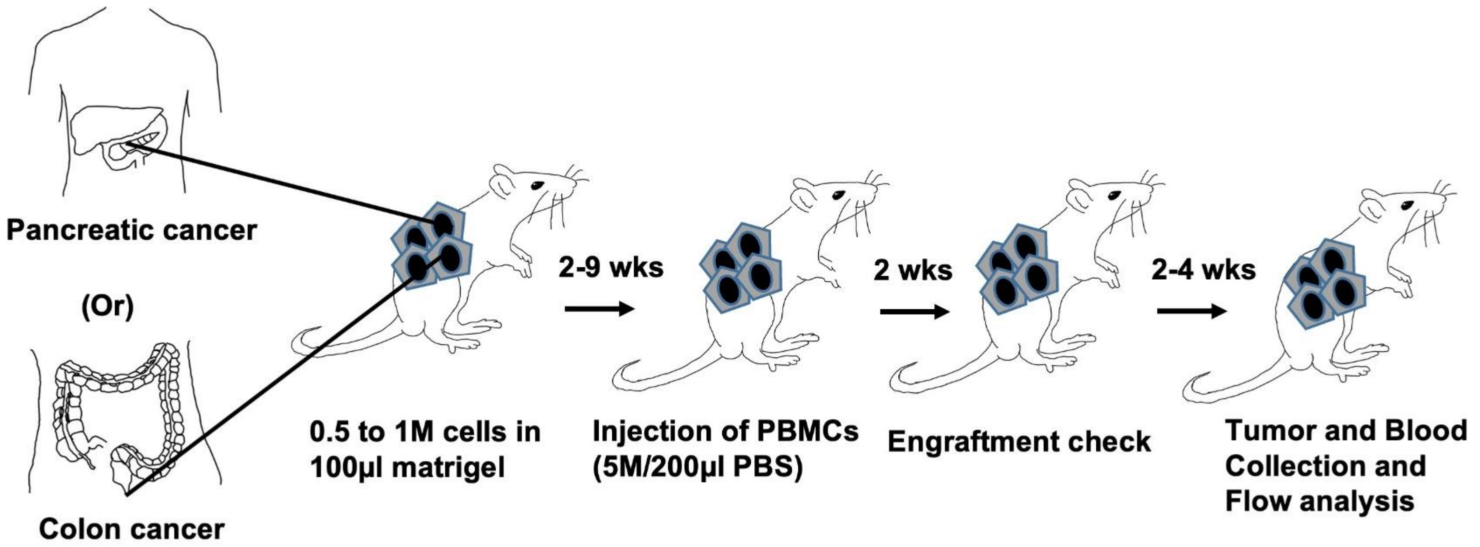

Pancreatic cancer
(Or)
Colon cancer
2-9 wks
2 wks
2-4 wks
0.5 to 1M cells in
100µl matrigel
Injection of PBMCs
(5M/200µl PBS)
Engraftment check
Tumor and Blood
Collection and
Flow analysis
