# Supplementary material for: Critical evaluation of an autologous peripheral blood mononuclear cell-based humanized cancer model
Source: PLoS One. 2022 Sep 12;17(9):e0273076. doi: 10.1371/journal.pone.0273076 (PMC9467357; doi:10.1371/journal.pone.0273076)
Supplement: S2 Fig — NSG mice (6-8-week-old) were injected with 5 million hPBMCs (i.v) and GVHD was documented over a period of 12 weeks. (A) GVHD scores (n = 9). (B) Average weight change and GVHD scores (n = 9). (C) Weight change in individual mice (n = 9). (PDF) [file pone.0273076.s002.pdf]

S2 Fig. PBMC demonstrate limited signs of GVHD for up to 8 weeks in immuno-compromised mice. NSG mice (6-8-week-old) were injected with 5 million hPBMCs (i.v) and GVHD was documented over a period of 12 weeks. **(A)** GVHD scores (n=9). **(B)** Average weight change and GVHD scores (n=9). **(C)** Weight change in individual mice (n=9).

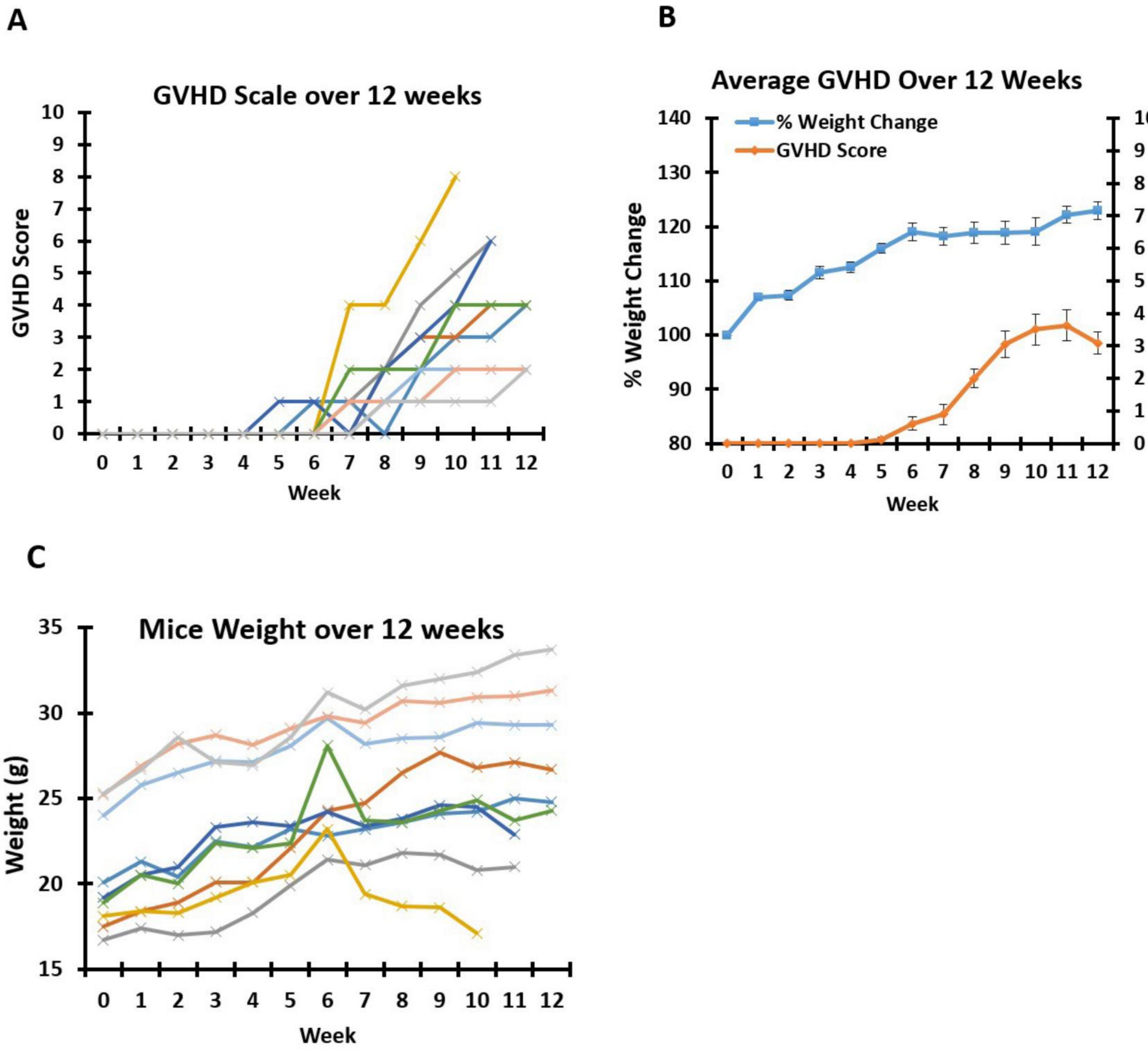
A
GVHD Scale over 12 weeks
GVHD Score
Week
0 1 2 3 4 5 6 7 8 9 10 11 12
B
Average GVHD Over 12 Weeks
% Weight Change
GVHD Score
% Weight Change
GVHD Score
Week
C
Mice Weight over 12 weeks
Weight (g)
Week
