# Supplementary material for: Critical evaluation of an autologous peripheral blood mononuclear cell-based humanized cancer model
Source: PLoS One. 2022 Sep 12;17(9):e0273076. doi: 10.1371/journal.pone.0273076 (PMC9467357; doi:10.1371/journal.pone.0273076)
Supplement: S3 Fig — (A) Representative flow cytometry plots are shown for MPC model CD3+CD45+ T lymphocytes from PDX mice (left), iPDX mice (middle). Data from samples collected at 2 weeks. (B) Bar graph of the average % of CD3+CD45+ cells (right). (C) Flow cytometry plots for CD4+ and CD8+ T lymphocytes from all iPDX mice (n = 3). Data from samples collected at 2 weeks. (D) The change in the average % of human T lymphocytes from early (2 week) to late (7 weeks) engraftment are shown for MPC CD3+CD45+ T lymphocytes (left), CD8+ T lymphocytes (middle) and CD4+ T lymphocytes (right). (E) PD-1+CD4+ T lymphocytes (left) PD-1+CD8+ T lymphocytes (right). T lymphocytes from healthy human donor blood (grey histogram, left) and iPDX blood (empty histogram, right). (F) Bar graph showing average percentage of PD-1+CD4+ and CD8+ T lymphocytes. Data from samples collected at 7 weeks. The values represent the percentage of human CD3, CD4 and CD8 population in iPDX mice peripheral blood. Representative averages of per group (n = 2) are shown. *P<0.05 and **P < 0.005. (PDF) [file pone.0273076.s003.pdf]

S3 Fig. Effective engraftment of human PBMC derived T cells in MPC iPDX mouse peripheral blood. **(A)** Representative flow cytometry plots are shown for MPC model $CD3^{+}CD45^{+}$ T lymphocytes from PDX mice (left), iPDX mice (middle). Data from samples collected at 2 weeks. **(B)** Bar graph of the average % of $CD3^{+}CD45^{+}$ cells (right). **(C)** Flow cytometry plots for $CD4^{+}$ and $CD8^{+}$ T lymphocytes from all iPDX mice (n=3). Data from samples collected at 2 weeks. **(D)** The change in the average % of human T lymphocytes from early (2 week) to late (7 weeks) engraftment are shown for MPC $CD3^{+}CD45^{+}$ T lymphocytes (left), $CD8^{+}$ T lymphocytes (middle) and $CD4^{+}$ T lymphocytes (right). **(E)** PD-$1^{+}CD4^{+}$ T lymphocytes (left) PD-$1^{+}CD8^{+}$ T lymphocytes (right). T lymphocytes from healthy human donor blood (grey histogram, left) and iPDX blood (empty histogram, right). **(F)** Bar graph showing average percentage of PD-$1^{+}CD4^{+}$ and $CD8^{+}$ T lymphocytes. Data from samples collected at 7 weeks. The values represent the percentage of human CD3, CD4 and CD8 population in iPDX mice peripheral blood. Representative averages of per group (n=2) are shown. $^{*}P<0.05$ and $^{**}P < 0.005$.

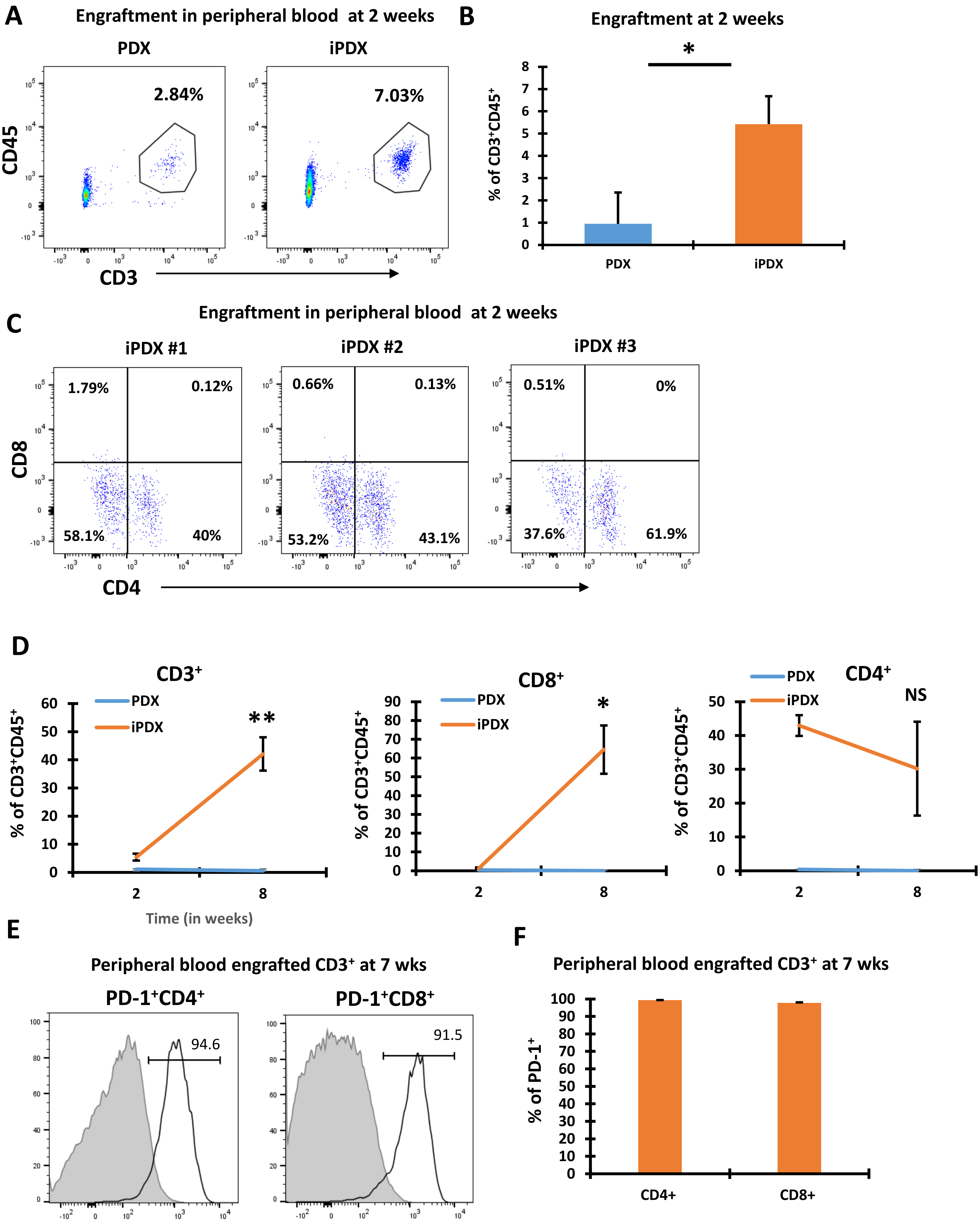
A
Engraftment in peripheral blood at 2 weeks
PDX
iPDX
2.84%
7.03%
CD45
CD3
B
Engraftment at 2 weeks
*
% of CD3+CD45+
PDX
iPDX
C
Engraftment in peripheral blood at 2 weeks
iPDX #1
iPDX #2
iPDX #3
1.79%
0.12%
58.1%
40%
0.66%
0.13%
53.2%
43.1%
0.51%
0%
37.6%
61.9%
CD8
CD4
D
CD3+
CD8+
CD4+
PDX
iPDX
**
*
NS
% of CD3+CD45+
Time (in weeks)
E
Peripheral blood engrafted CD3+ at 7 wks
PD-1+CD4+
PD-1+CD8+
94.6
91.5
F
Peripheral blood engrafted CD3+ at 7 wks
% of PD-1+
CD4+
CD8+
