# Supplementary material for: Critical evaluation of an autologous peripheral blood mononuclear cell-based humanized cancer model
Source: PLoS One. 2022 Sep 12;17(9):e0273076. doi: 10.1371/journal.pone.0273076 (PMC9467357; doi:10.1371/journal.pone.0273076)
Supplement: S4 Fig — (A) Representative flow cytometry dot plots showing CD3+CD45+ tumor infiltrating lymphocytes (TILs) (top left); (B) Bar graph of average values (top right). Data from samples collected at 7 weeks. (C) CD4+ and CD8+ TILs from 2 individual MPC iPDX mouse tumors (bottom panel). (D) PD-1+CD4+ TILs (left) and PD-1+CD8+ TILs (middle) from a representative MPC iPDX mouse tumor. T lymphocytes from non-cancer human donor PB obtained from phlebotomy lab (grey histogram, left) and TILs from iPDX tumor (empty histogram, right). (E) Bar graph showing average percentage of PD-1+CD4+ and CD8+ T lymphocytes from 2 individual MPC iPDX mouse tumors. Data from samples collected at 7 weeks. Values represent mean of two animals per group ± SD. **p < 0.005. (PDF) [file pone.0273076.s004.pdf]

S4 Fig. Identification and phenotypic characterization of pancreatic iPDX tumor derived human $CD3^+$ T cells. (A) Representative flow cytometry dot plots showing $CD3^+CD45^+$ tumor infiltrating lymphocytes (TILs) (top left); **(B)** Bar graph of average values (top right). Data from samples collected at 7 weeks. **(C)** $CD4^+$ and $CD8^+$ TILs from 2 individual MPC iPDX mouse tumors (bottom panel). **(D)** PD-1^+CD4^+ TILs (left) and PD-1^+CD8^+ TILs (middle) from a representative MPC iPDX mouse tumor. T lymphocytes from non-cancer human donor PB obtained from phlebotomy lab (grey histogram, left) and TILs from iPDX tumor (empty histogram, right). **(E)** Bar graph showing average percentage of PD-1^+CD4^+ and $CD8^+$ T lymphocytes from 2 individual MPC iPDX mouse tumors. Data from samples collected at 7 weeks. Values represent mean of two animals per group ± SD. $^{**}p < 0.005$.

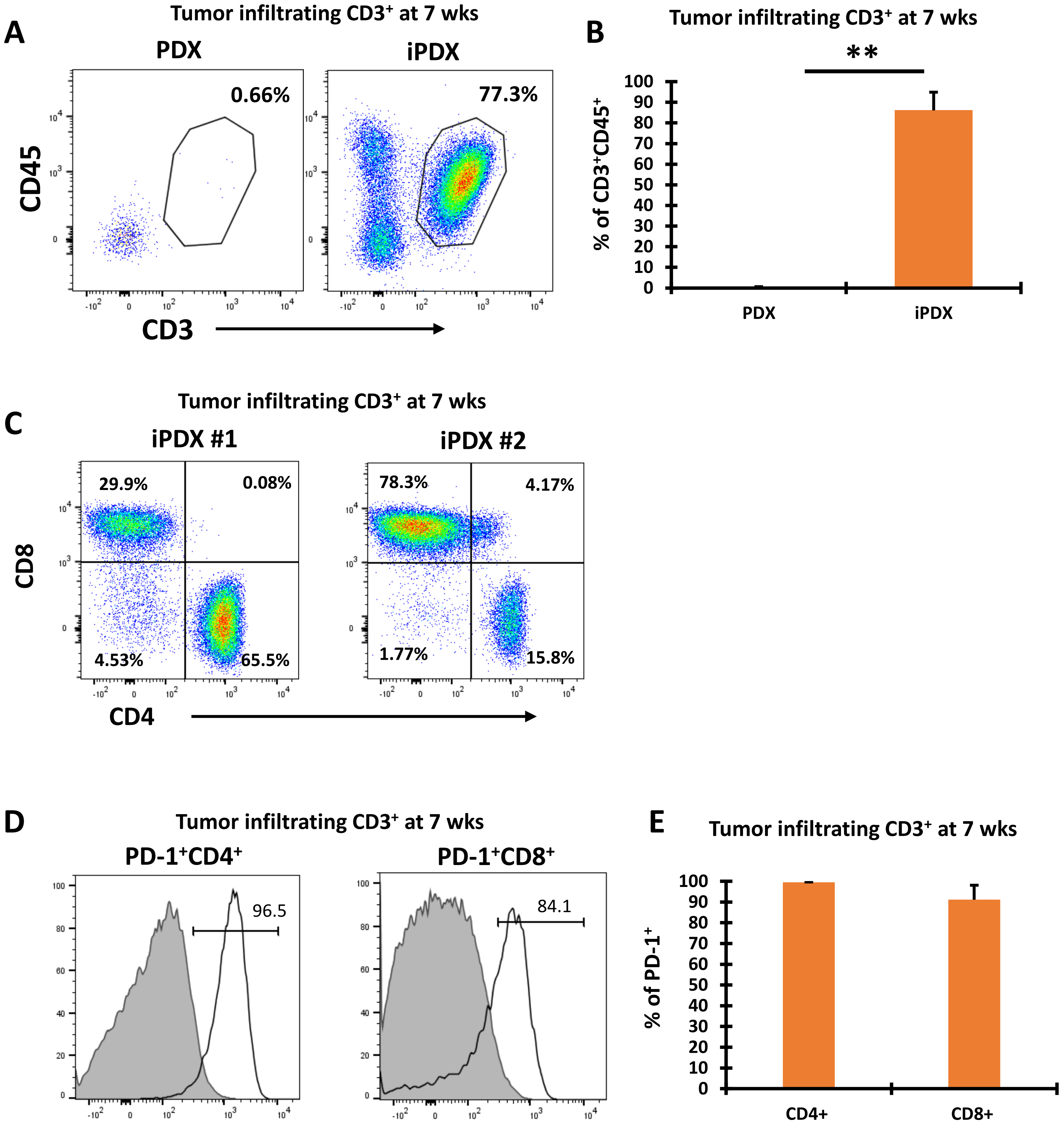
A
Tumor infiltrating CD3+ at 7 wks
PDX
iPDX
0.66%
77.3%
CD45
CD3
B
Tumor infiltrating CD3+ at 7 wks
**
% of CD3+CD45+
100
90
80
70
60
50
40
30
20
10
0
PDX
iPDX
C
Tumor infiltrating CD3+ at 7 wks
iPDX #1
iPDX #2
29.9%
0.08%
4.53%
65.5%
78.3%
4.17%
1.77%
15.8%
CD8
CD4
D
Tumor infiltrating CD3+ at 7 wks
PD-1+CD4+
PD-1+CD8+
96.5
84.1
E
Tumor infiltrating CD3+ at 7 wks
% of PD-1+
100
90
80
70
60
50
40
30
20
10
0
CD4+
CD8+
