# Supplementary material for: Critical evaluation of an autologous peripheral blood mononuclear cell-based humanized cancer model
Source: PLoS One. 2022 Sep 12;17(9):e0273076. doi: 10.1371/journal.pone.0273076 (PMC9467357; doi:10.1371/journal.pone.0273076)
Supplement: Attachment — Submitted filename: Response to Reviewers.docx [file pone.0273076.s006.docx]

E. Ramsay Camp, MD, FACS

Professor and Chief, Division of Surgical Oncology

**MICHAEL E. DEBAKEY DEPARTMENT OF SURGERY**

March 8, 2022

Anilkumar Gopalakrishnapillai
Academic Editor
PLOS ONE

Dear Dr. Gopalakrishnapillai,

Enclosed please find our revised manuscript entitled "**Critical evaluation of an autologous peripheral blood mononuclear cell-based humanized cancer model**" for consideration by *PLOS ONE*. We were pleased to be informed that the above-mentioned manuscript is of interest to *PLOS ONE*. We appreciate the reviewer’s comments and valuable input to improve our manuscript. We also appreciate that the manuscript was found to be technically sound and performed with rigor by both reviewers. In order to address the reviewer comments, we have revised and updated figures, as well, addressed weaknesses identified the comments. Comments from the editor and reviewers will be addressed individually in italicized font below.

**Editor Comments:**

*1. specifically would like to see authors including an immunotherapy modality in this model as a proof of principle to validate the work*.

We agree that it would be ideal to test an immunotherapy modality in our model to broaden our conclusions. In fact, this was the overarching goal as we stated in the introduction section. However, for the reasons outlined below, we concluded that a patient-derived autologous PBMC method for humanization may not be the optimal strategy to study cancer immunotherapy in a rigorous and reproducible manner. Thus, this manuscript is focused on our model development approach and the barriers that we encountered in optimizing experimental conditions.

Our goal was to develop a patient-derived humanized cancer mouse model based on the widely cited reference (*Sanmamed et al. Cancer Research, 2015*) that used a single gastric cancer patient tumor and PBMCs to study the use of Nivolumab and Urelumab. In retrospect, this elegant experiment only reported using a single patient sample and we could not find that it was repeated with the same patient samples or with another patient tissue samples. Our attempt to reproduce the published humanized model for immunotherapy testing with both colorectal cancer and pancreatic cancer tissue samples identified critical barriers that challenged the feasibility of the approach including:

- Early evidence of xGVHD in the xenograft prior to clinical xGVHD in the mouse.
- Significant intra- and inter model immune cell engraftment variability.

These unexpected observations ultimately led us to conclude that a PBMC-based humanized model would not result in a reproducible model system that reflects the human condition for testing cancer immunotherapy rigorously. The degree of variability was particularly concerning and challenged the feasibility of the model for immunotherapy testing. In addition, the limited PBMC supply obtained from cancer patients decreased our ability to repeatedly test models. To further emphasize the valuable points our investigations uncovered, we have revised the manuscript. We have now emphasized these barriers in the manuscript conclusions. On page 24 line 373, we revised the manuscript as “We ultimately concluded that the intra- and inter-model variability is a critical limitation towards developing a reproducible experimental system that can rigorously test immunotherapy strategies. Using larger sample sizes and selecting mouse models based on engraftment might be an option to overcome the variability.” In the abstract, we revised the final statements as “In summary, the iPDX models can reproduce key features of the corresponding human tumor. However, the observed variability in T cell engraftment and high PD-1 expression are important considerations that need to be addressed in order to develop a reproducible model system.”

While we were not able to achieve the original goal of our studies with the resources available for this effort, we believe that our manuscript will be very informative to future investigators who attempt a patient-derived autologous PBMC-humanized approach. On the surface, the model system is very appealing due to the relative ease of obtaining the needed patient samples. The issues we outline can provide future investigators with a realistic appreciation of the limitations and we expect the manuscript will serve as a vital reference for future investigators seeking to develop humanized mouse models for assessing immunotherapy.

**Reviewer’s comments and response.**

Reviewer #1:

Thank you for the comments that you believe our paper is “well written” and that you recommend for publication.

Reviewer #2

1. *Use of an immunotherapy modality in this model at least as proof-of-principle is lacking.*

Please refer to our comments to the Editor above. We completely agree and we were sincerely disappointed in the significant limitations that we encountered that were previously not reported or discussed. We hope that you appreciate our effort to reproducibly create an effective model and that our rigorous testing demonstrated critical limitations that we decided were important to report in order to inform future investigators.

2. *Fig. 3F How can the % of both CD4 and CD8 T cells be close to 80%? It is confusing as these markers are mutually exclusive.* Thank you for your comment. We intended to demonstrate the % of cell populations that were PD-1 positive in the xenografts. The total CD4 and CD8 T cell populations are demonstrated in 3D. To make figure 3F less confusing, we have bolded and enlarged the Y-axis title to make this clearer that it is only the %PD-1 positive cell populations in the analysis.

3. *Figures 1 and 2 should be put in the supplement.* We agree and now have moved these figures to the supplemental section and revised the figure legend accordingly.

Other requirements:

4. We have revised the Methods section to now include (1) methods of sacrifice (line 126), (2) methods of anesthesia and/or analgesia (line 130), and (3) efforts to alleviate suffering (line 137-138).

5. Funding statement has been updated and now includes the statement “There was no additional external funding received for this study.”

6. Funding Information’ and ‘Financial Disclosure’ sections now match.

Overall, we are grateful for the possible opportunity to publish our manuscript in *PLOS ONE.* We believe that our critical evaluation of a PBMC humanized cancer model system will provide important reference points for future investigators. Please do not hesitate to contact me should any questions arise. Thank you for your consideration in this matter.

Sincerely,

E. Ramsay Camp, M.D.
